# Supplementary material for: Landscape heterogeneity buffers biodiversity of simulated meta-food-webs under global change through rescue and drainage effects
Source: Nat Commun. 2021 Aug 5;12:4716. doi: 10.1038/s41467-021-24877-0 (PMC8342463; doi:10.1038/s41467-021-24877-0)
Supplement: Supplementary file 1 — Supplementary Information [file 41467_2021_24877_MOESM1_ESM.pdf]

Supplement:

Landscape heterogeneity buffers biodiversity of simulated meta-food-webs under global change through rescue and drainage effects

Remo Ryser, Myriam R. Hirt, Johanna Häussler, Dominique Gravel, Ulrich Brose

SUPPLEMENTARY METHODS

Supplementary Table 1: Parameters

Symbol	Parameter	Value
e_A	Conversion efficiency animal species	0.906[1]
e_P	Conversion efficiency plant species	0.545[1]
x_A<br>exp	Scaling constant and exponent metabolic rate animal species	0.141<br>-0.305[2]
x_P<br>exp	Scaling constant and exponent metabolic rate plant species	0.138<br>-0.25
c	Interference competition	0
a_0	Scaling factor capture coefficient for carnivorous links	15
a_1	Scaling factor capture coefficient for herbivorous links	3500
β_i; β_j	Allometric exponent for encounter rates	Carnivorous: 0.42; 0.42<br>Herbivorous: 0.19; 1 [3]
R_{opt}	Optimal consumer-resource body mass ratio	100
γ	Exponent Ricker's function	Foodchain: 2<br>Foodweb: 6
h_0	scaling factor handling time	0.4
η_i<br>η_j	Allometric exponent handling time (*i*: consumer, *j*: resource)	-0.48<br>-0.66 [4]
q	Hill coefficient	Foodchain: 0<br>Foodweb: 0.1
K	Half saturation density for nutrient uptake	Foodchain: 0.1<br>Foodweb: (0.1,0.2)
D	Nutrient turnover rate	0.25
S	Nutrient supply concentration	variable
d_{max}	Maximum dispersal distance	0.5

ε<br>δ_0	Scaling factor and exponent for species-specific dispersal distance	0.05<br>0.1256
a_S	Maximal emigration rate	Variable (Fig. 2b main text), 0.05
b	Shape parameter of emigration function	10 (0 for non-adaptive dispersal scenarios)
f	Additional scaling factor for capture rates for stability	0.05

SUPPLEMENTARY NOTES

Rescue effect

Increased dispersal loss (hostility) or the coupling with an oligotrophic patch (heterogeneity) essentially increases the strength of the drainage effect from the perspective of a eutrophic patch. However, while heterogeneity also increases the strength of the rescue effect from the perspective of an oligotrophic patch with a nutrient supply concentration of 1 (Supplementary Figure 1, left to right), dispersal loss decreases the strength of the rescue effect (Supplementary Figure 1, bottom to top) except at high heterogeneity where the pattern is slightly more complex. Here (Supplementary Figure 1, top-left), the weakened coupling with a eutrophic patch induces oscillations (see section on dynamical interference). Note that the sign on the heterogeneity axis is opposite compared to the main Figure Fig3b because here, it is the perspective of the oligotrophic patch that is coupled with a eutrophic patch, that then is reduced in its nutrient supply concentration (i.e. on the left side of the x-axis the nutrient supply of the oligotrophic (focal) patch is 1 and on the eutrophic patch it is 30, resulting in a heterogeneity of -29).

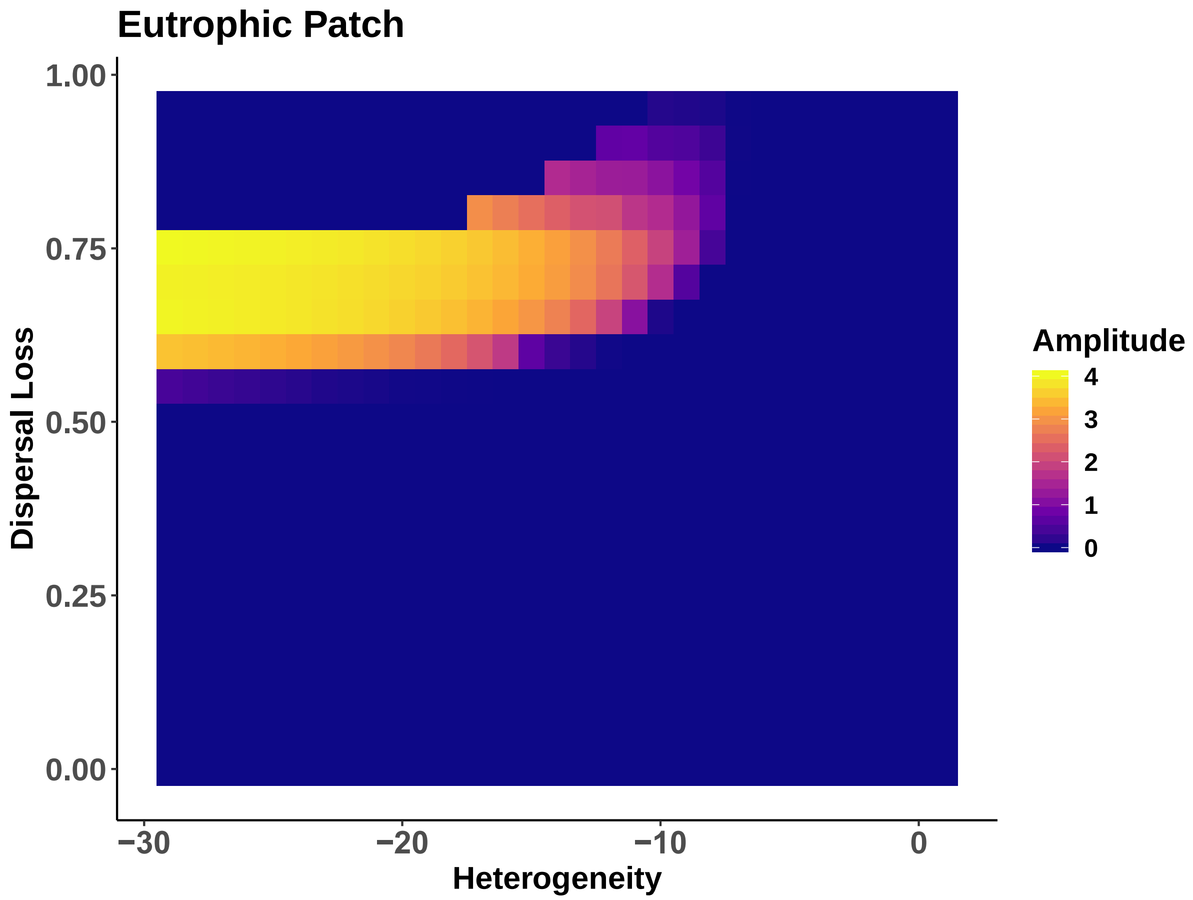

Supplementary Figure 1: Heat map showing the amplitude of biomass density oscillations in the predator (z-axis; colour coded) on the (always) oligotrophic patch across gradients of landscape heterogeneity (x-axis; difference in nutrient supply concentration between the two patches) and matrix hostility (y-axis) in a food chain on two patches. Amplitudes of 0 (blue) stand for an equilibrium state of the predator. Grey areas are where the predator went extinct.

Dynamical interference

When the hostility effect is very large, the coupling of the dynamics is weakened, which results in more chaotic oscillations as the frequencies get decoupled[5]. This in turn can lead to increased oscillations in the whole system that arise not from increased biomass fluxes but from dynamical interference (top quarter in Fig.3 and top-left corner in Supplementary Figure 1). This suggests that there is a lower threshold in strength of spatial links where instability arises from causes beyond the drainage and rescue effect.

This becomes apparent in the top four rows in Supplementary Figure 2. As soon as the frequencies get decoupled, the reduction of amplitudes due do the drainage effect is overwritten and amplitudes increase again on the eutrophic patch (red).

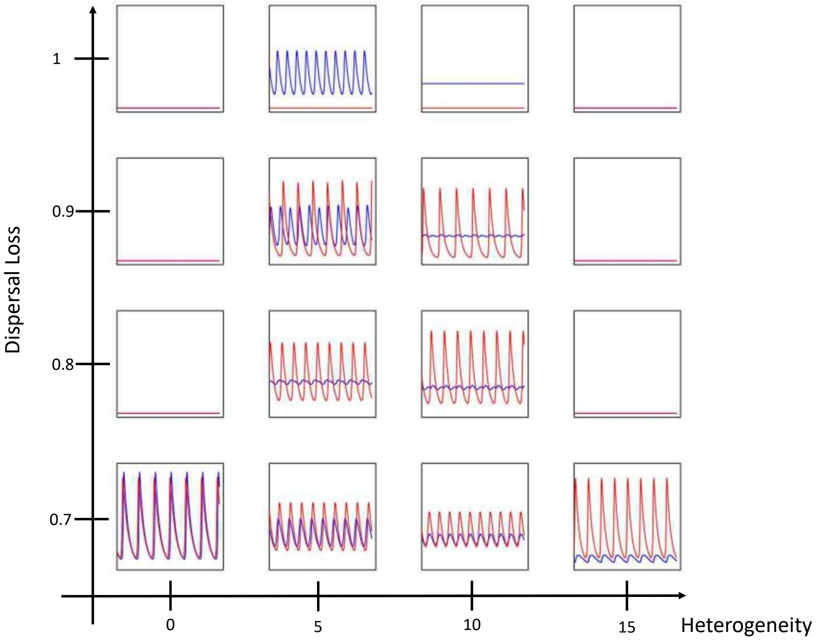
Dispersal Loss
1
0.9
0.8
0.7
0
5
10
15
Heterogeneity

50

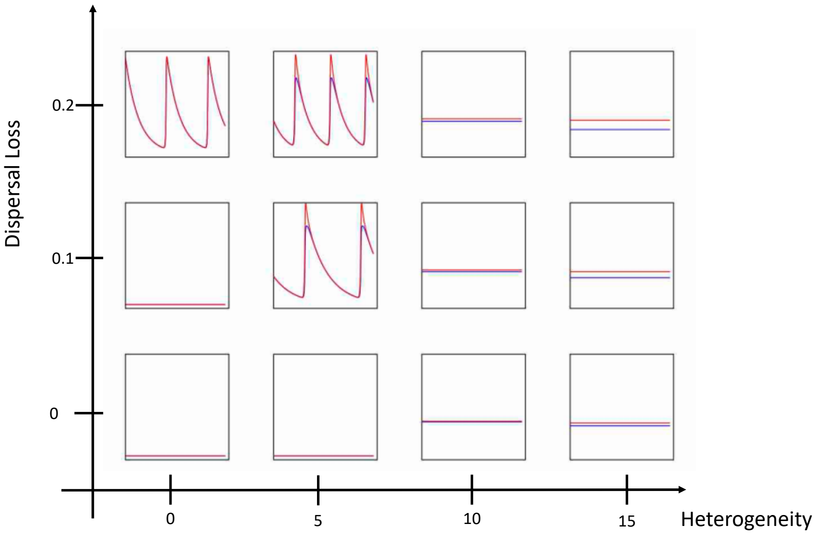
Dispersal Loss
0.2
0.1
0
0
5
10
15
Heterogeneity

51

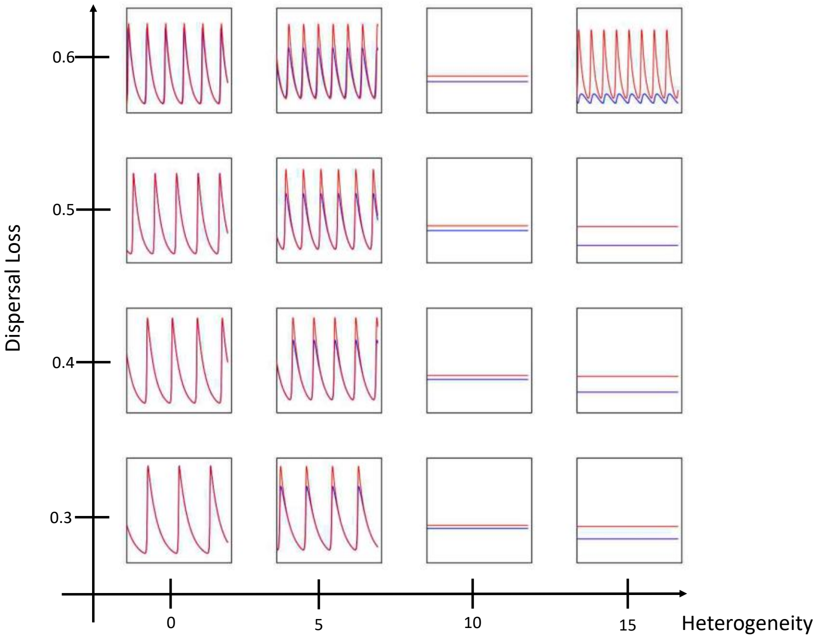

Supplementary Figure 2: Each plot represents biomass densities of the predator (y-axis) over time (x-axis) on the eutrophic patch (red) and on the variable patch (blue). Plots are arranged in a grid with the x-axis representing the landscape heterogeneity (delta nutrient supply of the eutrophic and the variable patch) and the y-axis representing the dispersal loss corresponding to Fig. 3 in the main manuscript.

Sensitivity

To test how strongly the drainage effect depends on the underlying dispersal model, we performed simulations in which the emigration rate is constant, i.e., is independent of local growth rates (referred to as non-adaptive dispersal – results presented in Supplementary Figure 3 and Supplementary Figure 4). In addition, we performed simulations with the adaptive dispersal model but with body mass independent dispersal ranges of organisms, i.e. organisms have the same dispersal range and therefore also the same dispersal success for a given interpatch distance. This means that all dispersing organisms experience the same dispersal loss rate and thus, the same matrix hostility (results presented in Supplementary Figure 5).

Non-adaptive dispersal

Increasing emigration rates ($d_{i,z}$ in equation 9; x-axis in Supplementary Figure 3), similar to an increasing maximum emigration rate (a in equation 10; x-axis in Fig. 2b in the main text), leads to a decrease in oscillation amplitudes of the carnivore population in a tri-trophic food chain on a single habitat patch.

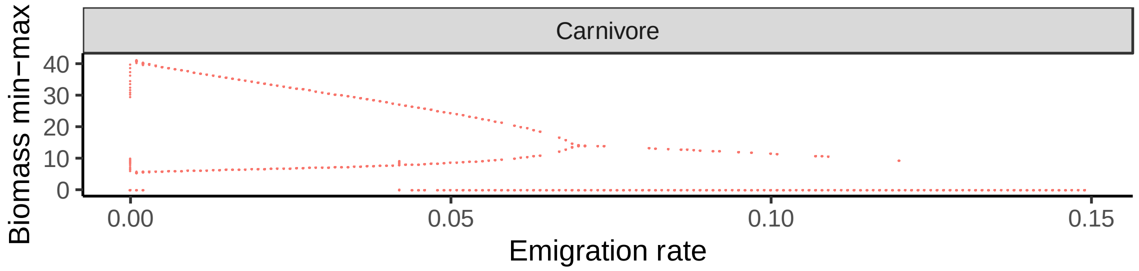

Supplementary Figure 3: Top-predator dynamics in a food chain on a single patch with increasing emigration rates and non-adaptive dispersal. The bifurcation diagram showing maximum and minimum biomass density (y-axis) when enabling emigration across a gradient of emigration rates (x-axis; $d_{i,z}$ in Equation 9) with a nutrient supply concentration of 10.

We repeated the simulations that produced the results presented in Fig. 3b in the main text with the non-adaptive dispersal model (results presented in Supplementary Figure 4). These simulations yielded almost identical results. This is also the case for the simulations in complex landscapes (compare Fig. 4 – adaptive dispersal with Supplementary Figure 5 – non-adaptive dispersal).

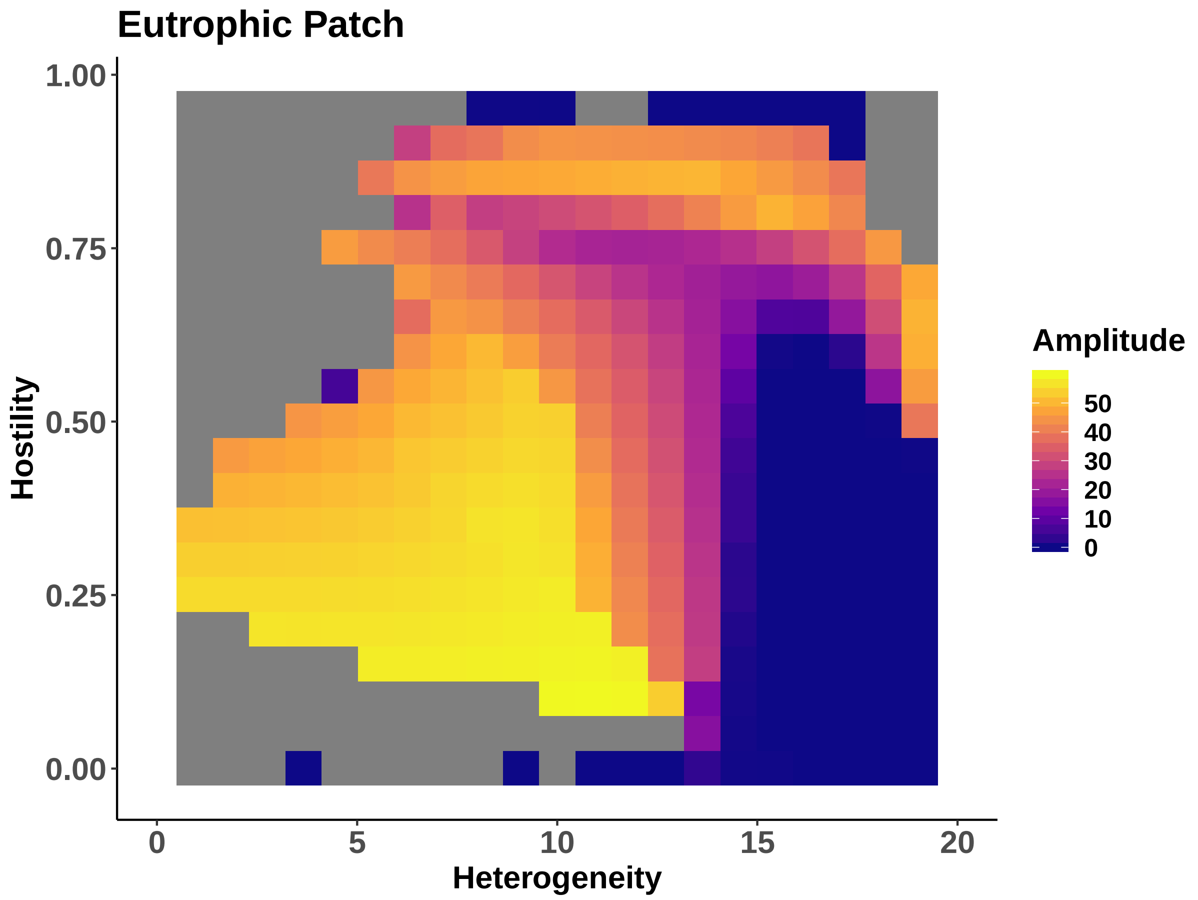

Supplementary Figure 4: Top predator dynamics of a tri-tropic food chain on two coupled patches with non-adaptive dispersal. Heat map showing the amplitude of biomass density oscillations of the predator (z-axis; colour coded) in the (always) eutrophic patch across gradients of landscape heterogeneity (x-axis; difference in nutrient supply concentration between the two patches) and dispersal loss (y-axis). Amplitudes of 0 (blue) stand for an equilibrium state of the predator. Grey areas are where the predator went extinct.

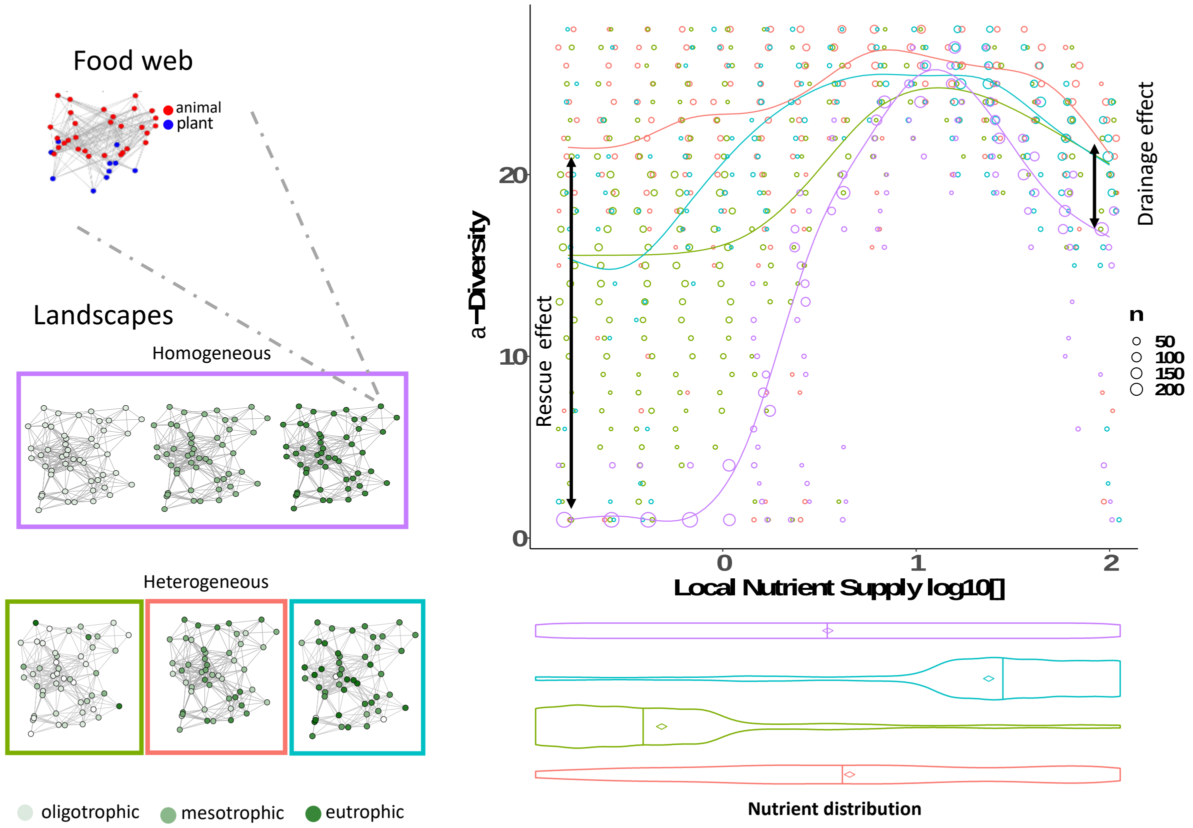

Supplementary Figure 5: Landscape heterogeneity drives biodiversity in complex meta-food-webs – non-adaptive dispersal. Local diversity on a patch (y-axis) across a gradient of local patch nutrient supply concentration in homogeneous (purple) and heterogeneous (green, orange, blue) landscapes. Violin plots below the x-axis show nutrient distributions within the landscape for each scenario, bars represent medians and diamonds represent means. The meta-food-web consists of a complex food web of 10 plants and 30 animals and large homogeneous and heterogeneous landscapes with 50 habitat patches with different patch nutrient supply concentrations (nutrient supply concentrations on habitat patches are colour coded). Edges indicate dispersal links for an exemplary species with a dispersal range of 0.3. Lines are a smooth fit from a GAM model with 95% confidence intervals in ggplot2, circles represent the data and the circle size the number of data points.

Non-body mass scaled dispersal range

To test the effect of species' body mass scaled dispersal range and resulting dispersal loss we repeated the simulations used for Fig. 3b in the main text and set the herbivore's dispersal range to be equal the carnivore's dispersal range. In the model used for the main results, the herbivore had a lower dispersal range compared to the carnivore due its smaller body mass. Thus, compared to the main results, the overall dispersal losses experienced by the herbivore and the carnivore is slightly lower here. Non the less, the results obtained from these simulations (presented in Supplementary Figure 5) remained very similar.

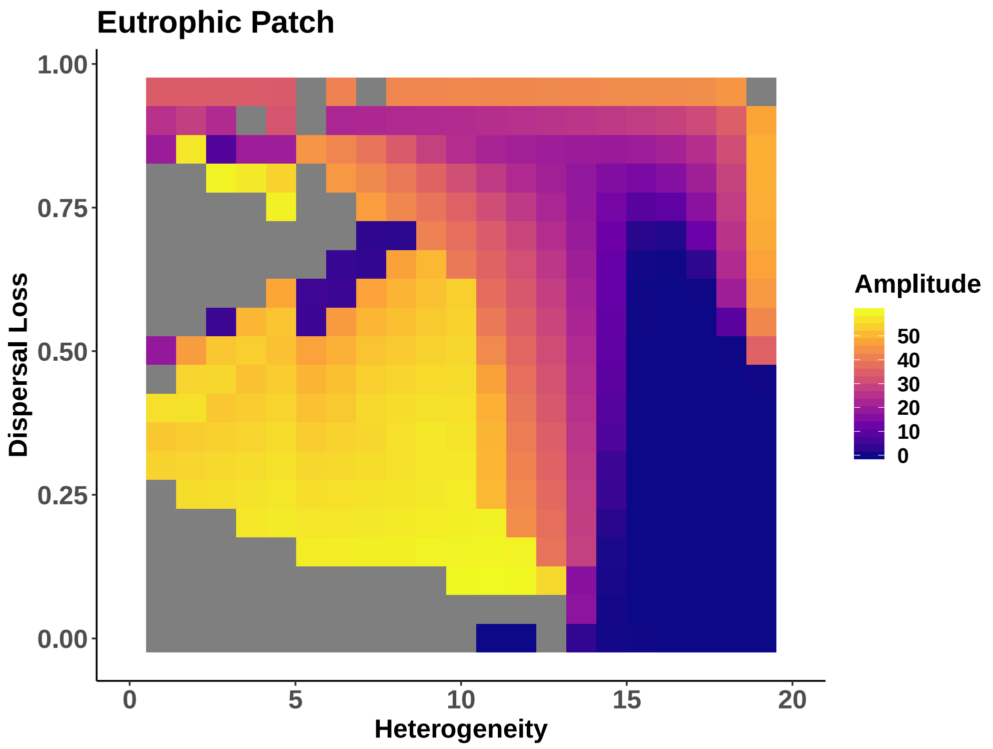

Supplementary Figure 6: Top predator dynamics of a tri-tropic food chain on two coupled patches with adaptive dispersal and non-body mass scaled dispersal range. Heat map showing the amplitude of biomass density oscillations of the predator (z-axis; colour coded) in the (always) eutrophic patch across gradients of landscape heterogeneity (x-axis; difference in nutrient supply concentration between the two patches) and dispersal loss (y-axis). Amplitudes of 0 (blue) stand for an equilibrium state of the predator. Grey areas are where the predator went extinct.

Keeping landscape-average of the nutrient supply constant

To address the effect of the landscape-average nutrient supply on top predator dynamics in a food chain on two dispersal-connected patches, we repeated the simulations that produced the results for Fig.3b in the main text but kept the average nutrient supply of both patches constant. This reduced the effect of heterogeneity and hostility from the perspective of a focal patch as the increase in drainage due to heterogeneity and hostility is counteracted by an increased local eutrophication.

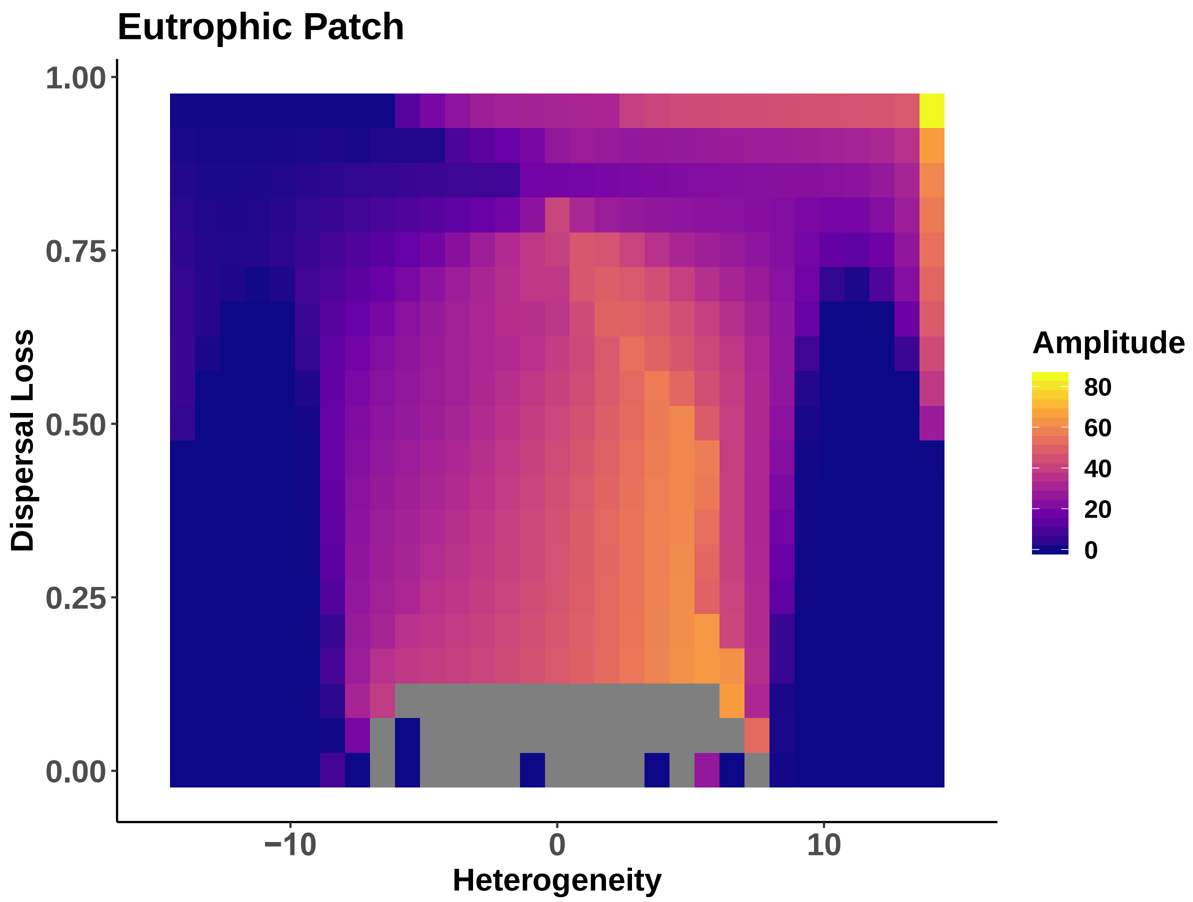

Supplementary Figure 7: Top predator dynamics of a tri-tropic food chain on two coupled with a constant landscape-average nutrient supply. Heat map showing the amplitude of biomass density oscillations of the predator (z-axis; colour coded) in the focal patch across gradients of landscape heterogeneity (x-axis; difference in nutrient supply concentration between the two patches) and dispersal loss (y-axis). Here, a heterogeneity of 10 corresponds to the focal patch having a nutrient supply that is 10 higher than on its neighbouring patch. Amplitudes of 0 (blue) stand for an equilibrium state of the predator. Grey areas are where the predator went extinct.

Plants and Animals separate

Splitting the results from Fig. 4 in the main text into plants and animals separately shows that it is mainly the animals profiting from rescue and drainage effects (Supplementary Figure 7) while plants only profit from the rescue effect (Supplementary Figure 8). Reasons for this may be, that increased animal diversity on oligotrophic patches in heterogeneous landscapes prevent competitive exclusion of plants, thus resulting in a cascading rescue effect. On eutrophic patches, however, the increasing drainage effect results in more animal species but may also decreases the biomass densities of animals, reducing their top-down effect on plants that eventually results in competitive exclusion of plants. Note that plants do not experience direct drainage and rescue effects as we do not let them disperse.

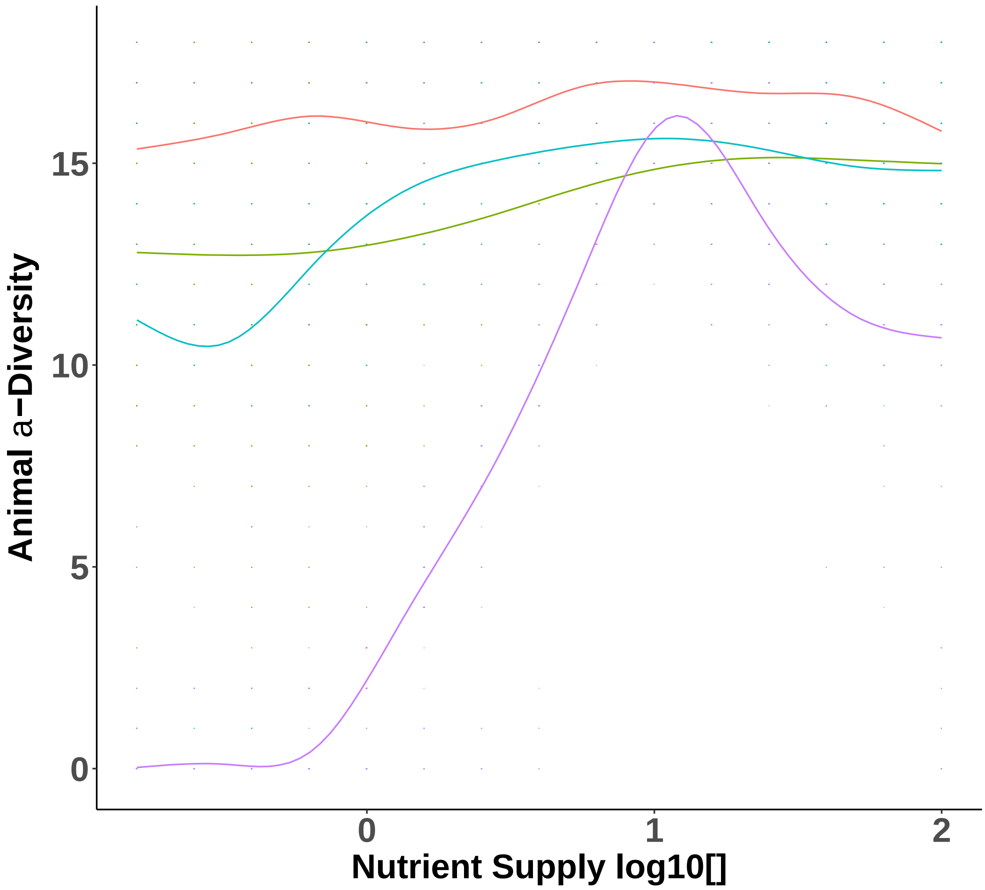

Supplementary Figure 8: Local animal diversity (y-axis) across a gradient of patch nutrient supply concentration in homogeneous (purple) and heterogeneous (green, orange, blue) landscapes.

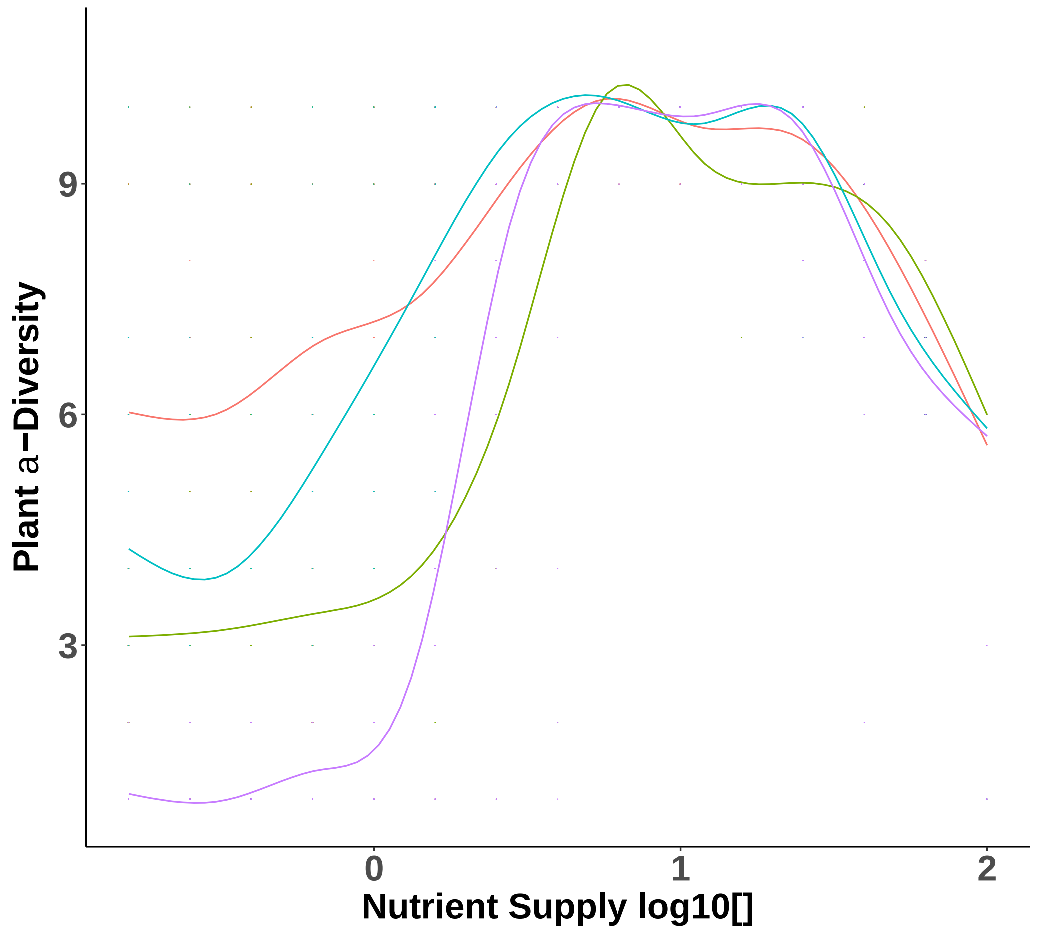

Supplementary Figure 9: Local plant diversity (y-axis) across a gradient of patch nutrient supply concentration in homogeneous (purple) and heterogeneous (green, orange, blue) landscapes.

SUPPLEMENTARY REFERENCES

1. Lang, B., Ehnes, R. B., Brose, U. & Rall, B. C. Temperature and consumer type dependencies of energy flows in natural communities. *Oikos* **126**, 1717–1725 (2017).
2. Ehnes, R. B., Rall, B. C. & Brose, U. Phylogenetic grouping, curvature and metabolic scaling in terrestrial invertebrates. *Ecol. Lett.* **14**, 993–1000 (2011).
3. Hirt, M. R., Jetz, W., Rall, B. C. & Brose, U. A general scaling law reveals why the largest animals are not the fastest. *Nat. Ecol. Evol.* **1**, 1116–1122 (2017).
4. Rall, B. C. *et al.* Universal temperature and body-mass scaling of feeding rates. *Philos. Trans. R. Soc. B Biol. Sci.* **367**, 2923–2934 (2012).
5. Blasius, B., Montbrió, E. & Kurths, J. Anomalous phase synchronization in populations of nonidentical oscillators. *Phys. Rev. E* **67**, 035204 (2003).
