# Supplementary material for: Landscape heterogeneity buffers biodiversity of simulated meta-food-webs under global change through rescue and drainage effects
Source: Nat Commun. 2021 Aug 5;12:4716. doi: 10.1038/s41467-021-24877-0 (PMC8342463; doi:10.1038/s41467-021-24877-0)
Supplement: Supplementary file 3 — Reporting Summary [file 41467_2021_24877_MOESM3_ESM.pdf]

nature research

Corresponding author(s): Ulrich Brose

Last updated by author(s): Jun 28, 2021

Reporting Summary

Nature Research wishes to improve the reproducibility of the work that we publish. This form provides structure for consistency and transparency in reporting. For further information on Nature Research policies, see our Editorial Policies and the Editorial Policy Checklist.

Statistics

For all statistical analyses, confirm that the following items are present in the figure legend, table legend, main text, or Methods section.

n/a	Confirmed	
x	☐	The exact sample size (*n*) for each experimental group/condition, given as a discrete number and unit of measurement
x	☐	A statement on whether measurements were taken from distinct samples or whether the same sample was measured repeatedly
x	☐	The statistical test(s) used AND whether they are one- or two-sided<br>*Only common tests should be described solely by name; describe more complex techniques in the Methods section.*
x	☐	A description of all covariates tested
x	☐	A description of any assumptions or corrections, such as tests of normality and adjustment for multiple comparisons
x	☐	A full description of the statistical parameters including central tendency (e.g. means) or other basic estimates (e.g. regression coefficient) AND variation (e.g. standard deviation) or associated estimates of uncertainty (e.g. confidence intervals)
x	☐	For null hypothesis testing, the test statistic (e.g. *F*, *t*, *r*) with confidence intervals, effect sizes, degrees of freedom and *P* value noted<br>*Give P values as exact values whenever suitable.*
x	☐	For Bayesian analysis, information on the choice of priors and Markov chain Monte Carlo settings
x	☐	For hierarchical and complex designs, identification of the appropriate level for tests and full reporting of outcomes
x	☐	Estimates of effect sizes (e.g. Cohen's *d*, Pearson's *r*), indicating how they were calculated

Our web collection on statistics for biologists contains articles on many of the points above.

Software and code

Policy information about availability of computer code

Data collection	Simulations were carried out using C++ and the SUNDIALS CVODE solver version 2.7.0. The code to reproduce the simulated data can be found at: https://github.com/RemoRyser/Metafoodweb
Data analysis	The simulated data were analyzed using R (version 4.0.5) and the package package ggplot2 (3.3.3).

For manuscripts utilizing custom algorithms or software that are central to the research but not yet described in published literature, software must be made available to editors and reviewers. We strongly encourage code deposition in a community repository (e.g. GitHub). See the Nature Research guidelines for submitting code & software for further information.

Data

Policy information about availability of data

All manuscripts must include a data availability statement. This statement should provide the following information, where applicable:

- Accession codes, unique identifiers, or web links for publicly available datasets
- A list of figures that have associated raw data
- A description of any restrictions on data availability

All data generated in this study can reproduced with the model code. The processed data can be found in the same github repository as the code: https://github.com/RemoRyser/Metafoodweb

Field-specific reporting

Please select the one below that is the best fit for your research. If you are not sure, read the appropriate sections before making your selection.

☐ Life sciences ☐ Behavioural & social sciences ☒ Ecological, evolutionary & environmental sciences

For a reference copy of the document with all sections, see nature.com/documents/nr-reporting-summary-flat.pdf

Ecological, evolutionary & environmental sciences study design

All studies must disclose on these points even when the disclosure is negative.

Study description	We simulated the population dynamics of species embedded in complex meta-food-webs. This involves their trophic interactions in local habitats and their dispersal dynamics across local habitats.
Research sample	For our analyses, we have been using all data that have been generated by simulations. For the simulations of complex food webs in complex landscapes, the simulations predicted alpha diversity for a total of 7500 patches.
Sampling strategy	All simulated data were used to illustrate the emerging patterns. Hence, there was no subsampling strategy.
Data collection	The simulations were carried out by Remo Ryser using self-written code in C++ (for availability of code see above).
Timing and spatial scale	The data have been created by simulations of C++ code. With the equations in the manuscript or the code (see above) they can be reproduced any time for any spatial scale (depending on model parameters).
Data exclusions	No data were excluded.
Reproducibility	The code for generating the data is publicly available which allows exact reproducibility.
Randomization	We started all simulations with random initial densities.
Blinding	Blinding was not relevant in our model study.

Did the study involve field work? ☐ Yes ☒ No

Reporting for specific materials, systems and methods

We require information from authors about some types of materials, experimental systems and methods used in many studies. Here, indicate whether each material, system or method listed is relevant to your study. If you are not sure if a list item applies to your research, read the appropriate section before selecting a response.

Materials & experimental systems

n/a	Involved in the study
☒	☐ Antibodies
☒	☐ Eukaryotic cell lines
☒	☐ Palaeontology and archaeology
☒	☐ Animals and other organisms
☒	☐ Human research participants
☒	☐ Clinical data
☒	☐ Dual use research of concern

Methods

n/a	Involved in the study
☒	☐ ChIP-seq
☒	☐ Flow cytometry
☒	☐ MRI-based neuroimaging
